# Validation Processes of Protein Biomarkers in Serum—A Cross Platform Comparison

**DOI:** 10.3390/s120912710

**Published:** 2012-09-18

**Authors:** Katja Köhler, Harald Seitz

**Affiliations:** Fraunhofer Institute for Biomedical Engineering IBMT, Branch Potsdam-Golm, Am Mühlenberg 13, 14476 Potsdam-Golm, Germany; E-Mail: Harald.Seitz@ibmt.fraunhofer.de

**Keywords:** clinical proteomics and diagnostic, multi-analyte immunoassays, serum screening, antibody-antigen interaction

## Abstract

Due to insufficient biomarker validation and poor performances in diagnostic assays, the candidate biomarker verification process has to be improved. Multi-analyte immunoassays are the tool of choice for the identification and detailed validation of protein biomarkers in serum. The process of identification and validation of serum biomarkers, as well as their implementation in diagnostic routine requires an application of independent immunoassay platforms with the possibility of high-throughput. This review will focus on three main multi-analyte immunoassay platforms: planar microarrays, multiplex bead systems and, array-based surface plasmon resonance (SPR) chips. Recent developments of each platform will be discussed for application in clinical proteomics, principles, detection methods, and performance strength. The requirements for specific surface functionalization of assay platforms are continuously increasing. The reasons for this increase is the demand for highly sensitive assays, as well as the reduction of non-specific adsorption from complex samples, and with it high signal-to-noise-ratios. To achieve this, different support materials were adapted to the immobilized biomarker/ligand, allowing a high binding capacity and immobilization efficiency. In the case of immunoassays, the immobilized ligands are proteins, antibodies or peptides, which exhibit a diversity of chemical properties (acidic/alkaline; hydrophobic/hydrophilic; secondary or tertiary structure/linear). Consequently it is more challenging to develop immobilization strategies necessary to ensure a homogenous covered surface and reliable assay in comparison to DNA immobilization. New developments concerning material support for each platform are discussed especially with regard to increase the immobilization efficiency and reducing the non-specific adsorption from complex samples like serum and cell lysates.

## 1. Why Is Candidate Biomarker Validation Important?

Validation of candidate biomarkers has a single goal: to determine, if there is sufficient evidence for a potential clinical utility of a given biomarker candidate to ensure further investment in that candidate for clinical trials. A validation is therefore essential before moving forward with this potential biomarker candidate. Thus, a clinical study is expensive, time-consuming, and has a high demand for clinical samples. Recently, a profound biomarker evaluation ensured the quality and clinical validity of a diagnostic assay [1]. Paulovich *et al.* dubbed biomarker validation as the tar pit, since the majority of biomarker candidates failed the clinical phase. Hence, the success rate of implementing a new biomarker candidate into clinical use is extremely low. However, plenty of publications about new biomarkers and diagnostic test platforms have been released within recent years. But, as the study of Fontela *et al.* revealed, published diagnostic tests for infection diseases often miss methodological quality and accurate reporting [2]. They used Quality Assessment of Diagnostic Accuracy Studies (QUADAS) and Standards for the Reporting of Diagnostic accuracy studies (STARD) tools for improving the reporting of diagnostic accuracy assays, regarding the quality of diagnostic studies in tuberculosis, malaria and HIV. Through systematic search of literature using PubMed and EMBASE (2004–2006), the sensitivity and specificity of various commercially available tests was compiled. Based on different quality items, they concluded that all studies had design deficiencies. For instance, merely 10% of the studies had an adequate description of the reference standard, less than 25% of the studies included a description of withdrawal, and none of these studies reported methods for the calculation and estimation of reproducibility.

Insufficient diagnostic test accuracy is just the tip of the iceberg. It is the consequence of poorly designed biomarker candidate discovery and validation phases without clear understanding of the nuances of interpreting high dimensional data sets which often leads to biases and high false discovery rates [3]. Many of these large list candidate biomarkers may discriminate between two classes of interest, but do not conform to the high standards of clinical trials. Moreover, candidate biomarkers that display the most significant differences between the cases and control group in the discovery dataset are often preferred without being tested, whether or not these are the most beneficial analytes for clinical decision making. Strategies which allow a high number of candidate biomarkers to be analyzed with the highest throughput and the lowest possible costs are required for an impartial validation. Based on the circulatory nature of blood through almost every parts of the human body, the measurements of blood components are particularly valuable for monitoring the health state of a person [4]. Certainly, proteomic-based biomarker discovery and validation directly in serum is challenging due to the complexity and the dynamic range of the analytes in plasma. The concentration range of serum proteins extends through eleven orders of magnitude, from albumin through cytokines [5]. Matters are complicated by the fact that potential candidate biomarkers are often present in low concentrations and are often bound to carrier molecules. Consequently, predominant high-abundance serum proteins may interfere and considerably influence the assay performance, as well as the quality of the result. This so called serum matrix effect, broadly defined as interference with the analytical technique by one or more components of the sample, can lead to loss of assay robustness, sensitivity and high levels of false positive and negative results [6].

The matrix effect is exceedingly relevant for mass spectrometry (MS) analysis, one of the principal enabling technologies for unbiased identification of novel target antigens. The milestone paper, that cemented the role of MS in clinical proteomics, was published by Petricoin *et al.* They identified components of the serum proteome by MS that allow a differentiation between patients with ovarian cancer from healthy individuals [7]. Numerous papers have been published that aimed to completely discover novel target antigens for diagnostic application by the help of MS [8–10]. However, insufficient analytical sensitivity of MS complicates the detection of low-concentration biomarkers within a complex mixture of high-abundance proteins [11]. Overcoming these detection limits is the biggest hurdle in MS analysis. Hence, the depletion of abundant protein fraction and the enrichment of biomarker are imperative to increase the sensitivity of the subsequent MS analysis [12]. Apart from the highly-abundant serum proteome, the discovery efforts alternatively focus on tissue or body fluids and moving to plasma once candidate biomarkers have been identified. Indeed, the protein composition of serum differs from that of tissue or body fluids. Serum protein depletion is a very time-consuming process, but the most critical issue is the accuracy and reproducibility of the analytical process caused by software problems. To increase the analytical reproducibility, MS labs typically run their samples 10 or more times, which implies a consumption of precious patient sample [13]. MS data helps to identify novel target antigens, but neither contains information about the applicability of the candidate in an immunoassay, nor the clinical validity.

A further strategy to identify novel antigens is via phage, bacterial, or mammalian cell-based cDNA libraries [14]. An important limitation of this approach is the lack of post-translational modifications, including glycosylation and phosphorylation. However, post-translational modified target antigens often contribute to diagnosis of autoimmune diseases like rheumatoid arthritis and systemic lupus erythematosus [15,16]. Post-translational glycosylation of antigens also represents an important target of anti-viral responses against HIV and influenza [17].

The current gold standard for biomarker validation and clinical diagnostics is the classical ELISA. Since this technique allows a relatively high-throughput and is a versatile and robust tool, ELISA is constantly employed for confirmatory studies. Thus, microarray analysis is often matched against the quantitative data of ELISA assays [18]. However, the ELISA is limited by the fact that the classic approach allows only a single antigen detection and often requires relatively large volumes of sample material compared with state-of-the-art methods. This concern becomes especially acute when clinical studies require access to limited amounts of biological material. Unfortunately, the ELISA performance strongly depends on the antibody quality as well as on the operator's skills and experiences. The solid nature of ELISA results in altering the immobilization of antigen to the solid matrix, which causes problems associated with accuracy and reproducibility. In comparison to other multiplexed immunoassays, ELISA has a relatively narrow dynamic range. This becomes particularly relevant when the biomarker of interest is detected in serum. Differences between samples are biased by comparison of serum that has biomarker levels within the dynamic range (does not require dilution in the assay) to serum above the dynamic range (does require dilution). The development of an ELISA assay is costly (typically, US$100,000–US$2 million per biomarker candidate) and is associated with a long development lead time (>1 year) and a high failure rate [19]. Compared with MS and traditional ELISA, multiplex arrays have several advantages, including their high-throughput nature, requirement for smaller sample volume, efficiency in terms of time and cost, the ability to evaluate one antigen in the context of multiple others, and the ability to reliably detect different proteins across a broad dynamic range [20]. Multi-analyte immunoassays might be the tool of choice for the identification and detailed validation of protein biomarkers in serum.

## 2. Multi-Analyte Immunoassays

**Figure 1.**
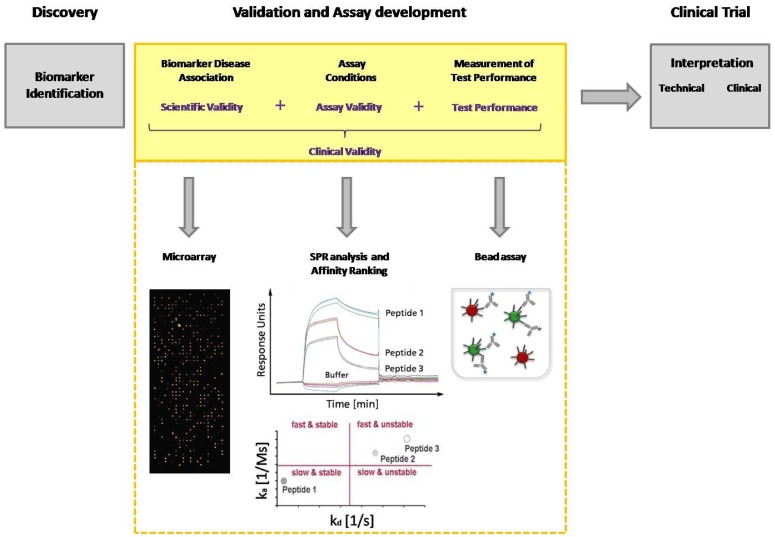
The process of candidate biomarker identification and validation.

Today, immunoassays are the fundamental method of protein analysis and have gained importance as powerful tools in basic and applied proteomic research. Immunoassays have their origin in the 1950s, when Berson and Yalow developed the first immunoassay for insulin, using radioactive labeled antibodies for detection [21]. To avoid radioactivity, in 1971 Engvall and Perlmann introduced an alkaline phosphatase conjugated antibody for detection that displays the classical detection principle of an ELISA (Enzyme-linked immunosorbent assay) [22]. In the late 1980s, Ekins, the founder of multi-analyte immunoassays, conceived the “Ambient-Analyte-Theory” and discussed the application of a parallelized microspot immunoassay in immune diagnostics [23,24]. His theoretical examinations and his experimental work demonstrated that simultaneous microspot immunoassays can be carried out with classical ELISA. Through miniaturization, a greater sensitivity and selectivity, as well as a higher throughput can be achieved. However, Ekins' theory gained even more importance after the development of DNA chip technology in the 90's, which coincided with the worldwide breakthrough of biochip technology [25]. The innovation of advanced equipment such as pipette robots, spotters and fluorescence readers allowed the production of microarrays with thousands of microspots, as well as the sensitive and high-resolution detection of bound target molecules. Since DNA microarrays are a well established tool in molecular biology, several groups have used this technology in order to immobilize proteins instead of DNA on the arrays [26,27]. Those arrays are miniaturized immunoassays used for protein or antibody detection from complex media. The rapid rise of immunoassays in protein analytics, combined with the development of protein arrays, and later peptide arrays as multiparametric immunoassay, illustrates the enormous potential of this technique in diagnostics. Within the last decade, multi-analyte immunoassays have led to the identification of a huge number of biomarkers from serum, particularly new antigens, and to the elucidation of disease progress. However, the current limitation is the validation of biomarker candidates. One of the major challenges in biomarker validation and diagnostic assay development is the availability of high-quality capture molecules, e.g., antibodies. Several efforts have been seek for the assortment of well-characterized affinity-based molecules, not limited to antibodies, in one database [28,29]. But also different other aspects of the entire validation process need to be improved. Technologies like MS and ELISA currently used for biomarker discovery and validation are not able to achieve the combination of high throughput with high measurement accuracy and precision. Compared with traditional ELISA, multiplex arrays have several advantages, including their high-throughput nature, requirement for smaller sample volume, efficiency in terms of time and cost, the ability to evaluate one antigen in the context of multiple others and the ability to reliably detect different proteins across a broad dynamic range. Multi-analyte immunoassays are excellently suited for the entire biomarker discovery process. During the validation procedure biomarkers must be identified and the disease association must be explained as well as the assay conditions must be defined before moving with that candidate in clinical trial (Figure 1). Hence, a clinical study is expensive, time-consuming, and a consumption of rare patient samples should be prevented. The group of Peter Nilsson pointed out the potential of staged proteomic profiling of serum by using a combination of different affinity-based methods. They had also considered the primary problem of biomarker analysis: the establishment of efficient throughput methods for validation of large generated datasets. Larger studies are needed to investigate the protein expression with the help of independent microarray platforms, and to finally overcome the inherent throughput limitations of Western Blot and ELISA. This dual confirmatory approach allows large-scale cross platform analysis and validation of antibody performance under assay conditions [30].

The evaluation of clinical validity and utility is imperatively needed. The process of identification and validation of serological markers as well as their application in diagnostic routine requires a combination of independent immunoassay platforms with the possibility of high-throughput measurements. This paper focuses on microarrays, bead assays and array-based surface plasmon resonance (SPR) assays and discusses their application in serum biomarker discovery.

The process of candidate biomarker validation has to be improved by using an approach comprising different affinity-based methods with the possibility for high-throughput. Rapid screening of interactions and biomarker disease association is carried out with microarrays. Label-free methods like surface plasmon resonance (SPR) are strongly recommended for the in-depth validation of these interactions. This analysis provides data about association and dissociation phases, as well as affinity constants. With the help of an affinity ranking, the interactions are classified according to their properties. Kinetic parameters and the kinetic classification allow the development of fine-tuned diagnostic assays concerning incubation time and washing stringency. Both strongly contribute to the implementation of candidate biomarkers into a new diagnostic assay. The defined assay conditions and performance of the candidate biomarkers can be tested in a bead assay.

## 3. A Cross Platform Comparison

Microarrays are miniaturized biological devices within which capture probes are fixed in defined positions on a supporting material, normally a standard sized glass slide. The analyte bound to the capture probe is detected by fluorescence. Microarrays made Ekin's concept of a miniaturized bioanalytical assays a reality. They allow many parameters to be tested at the same time under different condition. With parallelization in mind, the microarray can be divided into several subarrays. This makes the parallel screening of thousands of capture probes in one experiment possible. Such microarrays are ideally suited for an unbiased and rapid identification of a host of antibody-antigen interactions. However, problems including immobilization efficiency, fluorescence detection, data analysis, and signal-to-noise ratio (S/N ratio) must be critically addressed to improve the assay performance [31].

Glass slides often function as solid supports on which thousands of different capture molecules can be immobilized. Since tiny amounts of sample volume (reaching up to several nanoliters) per microspot are deposited, special liquid handling robots are required. Those are often constructed for the purpose of the DNA microarray development. Nevertheless, the spotting of purified proteins or peptides is much more challenging. They are more chemically diverse than DNA and their stability and functional nature on the array have to be ensured. An alternate approach to spotted proteins is the Nucleic Acid Programmable Protein Array (NAPPA) that circumvents the technical limitations of protein spotting. It is constructed by spotting protein-encoding plasmid DNA on an array surface and the proteins are subsequently generated using cell-free expression systems. Thus, the NAPPA platform allows functional protein studies including protein-small molecule, protein-protein, protein-nucleic acid, and also antigen-antibody interactions [32]. NAPPA microarrays were used to detect autoantibodies in human sera of breast cancer patients to p53 antigen and to GAD65 antigen in type 1 diabetes patients [33]. Dependent on the microarray application, several covalent or non-covalent immobilization strategies for capture probe binding are established (Table 1). These microarray substrates have to fulfill two major requirements to achieve maximal immobilization efficiency: It is important that the substrates provide high binding capacity and preserve the capture probe in an active state [34].

**Figure 2.**
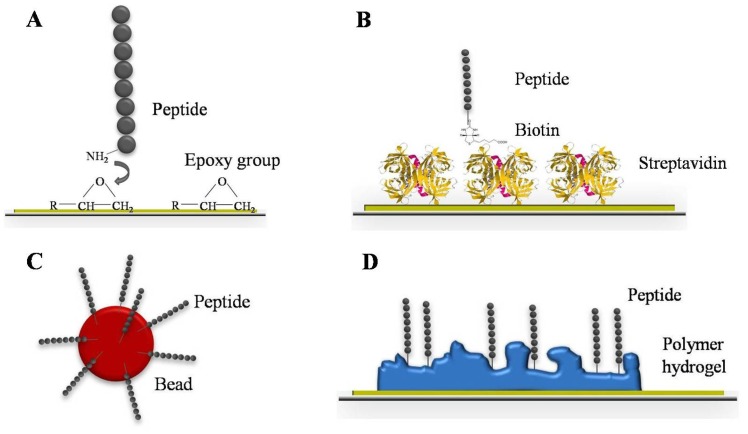
Schematic overview of the common surface molecular architectures. Functional groups such as epoxy (**A**) or a simple streptavidin layer (**B**) represent a 2-dimensional surface architecture. In comparison 3-dimensional surfaces including fluorescent microspheres (**C**) and polymer hydrogel layers (**D**) exhibit a higher capacity for capture probes.

On aldehyde, epoxy or amine coated substrates every functional group of the protein can react with the reactive group on the surface, therefore proteins and peptides are immobilized randomly. Thus, chemically or biologically active domains are sometimes not accessible for the analyte. For directional immobilization, the capture probe has to be chemically functionalized to differentiate the domain available for immobilization from those of chemical/biological activity. This leads to higher signal intensities and improved S/N ratios [36]. Schulze *et al.* demonstrated that special amino acids such as histidine and tyrosine at the *N*-terminus of the capture probes resulted in an improved immobilization efficiency on epoxysilane surfaces and higher signal intensities [37]. Ethanolamine that reacts with the epoxy group and does not preferably cover the capture probe has been used as blocking solution for epoxy surfaces. Thus, the S/N ration is additionally increased. Epoxy and other functional group substrates constitute two-dimensional coatings since the capture probe is in direct contact with the activated glass surface. Alternatively to epoxy, the capture probe can also be a part of simple monolayer architecture. Andresen *et al.* prepared a monolayer mixture of streptavidin and biotinylated peptides for site-specific immobilization on aldehyde coated glass slides in a two-step procedure (Figure 2B) [38]. This robust peptide microarray platform allows serological diagnosis of infection diseases. Beside these two-dimensional surfaces, three-dimensional coatings were developed as well (Figure 2D). The three-dimensional space of these polymer-based hydrogels leads to a higher capacity for capture probes. More molecules can be immobilized onto a limited space [39]. Higher amounts of immobilized capture probes results in increased total signal intensities and assay sensitivity. Since polymers self-adsorb onto the glass surface when simply immersed in aqueous solution, the coating procedure is fast and inexpensive. To improve the microarray robustness and reproducibility, Schröder *et al.* focused on the implementation of dual-color read-out for antibody microarrays [40]. Protein samples such as plasma, serum, urine, tissue, or cell culture can be analyzed without prior expensive and elaborate protein depletion steps. Depending on the study, there are two different experimental design options. For a direct comparison, two different sample types are labeled with different fluorescent dyes and competitively incubated on the same array. As an alternative a reference-based design is possible. Thereby, all protein samples are labeled with the same fluorescent dye and competitively incubated with a common reference, which is labeled with the second fluorescent dye. The evaluation of signal intensities is carried out by using the ratio of the two color channels for identification of differences between the samples. Technical variation effects are abolished by considering the ratio of the signal intensities. This leads to higher reproducible data, sensitivity, and consequently improved assay robustness. Overcoming the practical hurdles, microarrays represent a sensitive screening tool for serum biomarker discovery and have the capacity for an unbiased approach. Analyzing the IgE reactivity in patient sera, Hiller *et al.* used a microarray composed of 94 immobilized purified allergen molecules that represent the most common allergen sources [41]. This allergen microarray allowed the determination and monitoring of patients' IgE reactivity profiles and the identification of allergy eliciting molecules. In order to characterize the antibody response of distinct human autoimmune disorders including systemic lupus erythematosus and rheumatoid arthritis, Robinson *et al.* screened different patient sera by the help of peptide microarrays [42]. Thereby, the specificity and pathogeneses of autoantibodies was elucidated and new autoantigens were identified. In serum diagnostics, peptide arrays are well-established tools for antibody response screening. Peptides are only present as continuous epitopes. Nevertheless, peptides have many advantages compared to native protein antigens [43]. The synthesis of peptides is chemically established and completely automated. Based on their short length (10–15 amino acids), peptides hardly build up a secondary or tertiary structure which makes them chemically and physically more robust than proteins. Furthermore, diverse covalent and non-covalent immobilization strategies of peptides, up to the direct synthesis on solid support, are known and established [44]. In addition to their application as screening tools of autoimmune disorders, peptide microarrays have been successfully used for the detection of microbial infection diseases. Andresen *et al.* developed a peptide microarray consisting of viral peptides to demonstrate the specific binding of monoclonal antibodies [45]. In this regard, they were able to detect the binding of commercial available antibodies up to picomolar concentrations. That demonstrates the potential of microarrays, screening for antibody subtypes which are usually present in low concentrations like IgE. Similarly, bacterial infections like tuberculosis caused by *Mycobacterium tuberculosis* can be diagnosed. Using peptide microarrays, Nahtman *et al.* identified novel antibody epitopes and characterized the antibody reactivity profile on bacterial antigens [46]. However, if there is a lack of known antigens, random sequence peptide libraries can be used for serum screening [47]. In this manner, the antibody repertoire binding pattern of two different mice strain sera—healthy and infected with *Heligmosomoides polygyrus*, an intestinal helminthic parasite—were analyzed. Due to the library design and sophisticated microarray data analysis, a classification between healthy and infected mice could be predicted with a small number of peptides. Besides planar microarrays, robust and flexible bead assay systems have been developed over the last decade. The design of a bead-based immunoassay is comparable to that of a microarray: the capture probes are likewise immobilized on a solid support—beads. In this case, the capture probe is bound to microspheres and incubated with the analyte in suspension, which allows an improved mixing procedure and automation (Figure 2C).

**Figure 3.**
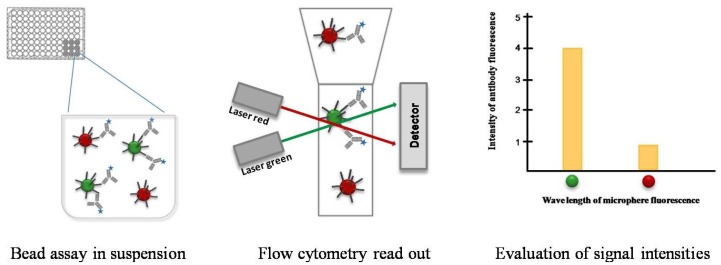
Schematic description of the bead assay.

These microspheres can consist of a variety of different materials, like color-coded beads or magnetic particles with different diameters. In comparison to location coded microarrays, whose capture probes are printed in picoliter volume pointwise onto the supporting material, a liquid handling robot is not imperatively needed for bead assays. Since bead assays are usually performed by using standard laboratory equipment, *i.e.*, using microtiter plates and a flow cytometer. The individual capture probes on the microarrays are detected as illuminated spots, depending on their location on the array surface. In the case of bead-based systems, the differentiation of capture molecules occurs with a color-coding of the beads. The color coding of the beads offers the potential of multiplexing. Depending on the number of different colors, each bead population can be coated with another capture probe that allows the binding and detection of specific analytes from a serum sample. A mixture of color-coded beads that have bound the antigen are incubated with a serum sample in a cavity of a microtiter plate (Figure 3). Serum antibodies with the corresponding paratope recognize and bind to the antigen. The higher the concentration of corresponding antibodies in the sample the more antibodies can bind to the beads. The detection of the bead-bound antibody-antigen-complex occurs with help of secondary fluorescent-conjugated antibodies. The spectral range of the antibody fluorescent dye differs from this of the internal microsphere dye. Thus, the classification of the beads (red, green) and the quantification of the signal intensities caused by the detection of antibody-antigen complex (blue) can be simultaneously read out by a flow cytometer. The outcome of the multiplex antibody-antigen reactions are displayed fully automated in the analysis software. The Luminex xMAP technology has become the most popular bead assay platform and belongs to one of five assay platforms available to serologically diagnose the West Nile Virus infection [48]. This bead assay consists of 100 different bead populations, each of them with two fluorescence dyes in 100 different coding ratios which are used for multiplexing. Via the fluorescence coding of the bead populations, a specific labeling is assigned for every immobilized molecule species.

Another platform, the BD FACSArray Bioanalyzer, is based on flow cytometry measurements. The differentiation between different bead populations occurs over a range of bead sizes and two fluorophores, which increases the degree of assay complexity. Bead assays enable the analysis of different capture probes within one reaction tube such as the cavity of a microtiter plate. In clinical diagnostics, these plates have become a usual lab standard and allow an automated high-throughput treatment of patient samples. Due to the easy handling and flexibility of the assay conditions, many commercially available immunoassays for the detection of allergies, autoimmune diseases, and infections are bead based. Earle *et al.* developed a fluorescent multiplex array for detecting six common indoor allergens in house dust samples [49]. This assay consisted of monoclonal antibodies that were covalently coupled to fluorescent microspheres of the Luminex xMAP platform. These antibodies have been well-defined and have a high specificity for their epitopes, a fundamental requirement for developing a reliable assay. The same group also studied intensely the crystal structures of allergens, as well as the interaction between these allergens with their antibodies [50–53]. The assay performance, including sensitivity, detection limit, and reproducibility was compared with an ELISA method, the gold standard for environmental exposure assessment. The determined intra-assay reproducibility resulted in a CV of less than 10% and in most cases the inter-assay CVs of the multiplex bead assay (>15%) were found to be slightly better than that of a normal ELISA. By doing a multiplex bead assay the dynamic range of the measurement was increased and allowed each sample to be tested at only two dilutions (1:100 and 1:10,000) to cover the full range of allergens. Compared to normal ELISA the detection limit was an order of magnitude lower for the bead assay and decreased to less than 1 ng/mL for each of the allergens. In this regard, a greater assay efficiency and reproducibility was achieved by measuring six or more allergens simultaneously in a single microtiter well. This has led to several technical improvements compared to normal ELISA. Currently, the presented multiplex array is commercially produced and sold by the company Indoor Biotechnology Inc. The number of measured antigens has been increased allowing the simultaneous detection and quantification of eight common indoor allergens in house dust samples. This technology represents the state-of-the-art technology in allergen detection.

Abreu *et al.* used the FIDIS™ technology by Biomedical Diagnostics for simultaneously detection of autoantibodies to diagnose the chronic autoimmune disease rheumatoid arthritis [54]. The FIDIS™ technology combines the Luminex xMAP^®^ platform with a flow cytometer, and includes a software for data analysis. This offers technological opportunity in laboratory bench top integration and automation. The antigens, human and animal IgG Fc fragments, were covalently bound to different sets of microspheres and incubated with patient serum samples. The autoantibody levels, measured with FIDIS™, were compared with routine laboratory tests regarding the clinical sensitivity and specificity. The results demonstrated the efficiency of FIDIS™ with an analytical performance equivalent to conventional methods such as ELISA and latex agglutination test. Nevertheless, the important benefits of FIDIS™ are the time and cost-saving measurements and the use of minimal sample volumes.

Bead assays are also used for the detection of arising infections. Binnicker *et al.* applied the BioPlex 2200 system that was developed by Bio-Rad Laboratories in cooperation with Luminex for the serological diagnosis of primary acute Epstein-Barr virus (EBV) disease [55]. The EBV diagnosis requires a combination of several antibodies for a reliable prediction. Thus, multiplex immunoassays represent an appropriate technology for identification of multiple antibody populations in a single reaction and thereby expediting detection of EBV. The serological assay was based on the simultaneous detection of five different IgG and IgM class antigens. The EBV viral capsid antigen (VCA), nuclear antigen-1 (NA), and early antigen-diffuse (EA-D) represent IgG epitopes, whereas heterophile antibodies and IgM-VCA were IgM derived. The serological response to EBV, obtained with the BioPlex 2200 system, was comparable with the results of conventional methods used to detect EBV specific antibodies. Thus, the assay yielded the same sensitivity and specificity as routine tests such as indirect immunofluorescence assay and enzyme immunoassay. However, the interpretation of data, and a confidential diagnosis appears to be crucial, and several groups have dealt with this issue. In conclusion, BioPlex 2200 EBV assay represents a valid evidence-based tool but, should be used in association with other laboratory and clinical parameters for classification of EBV disease state [56,57].

Similar to microarrays, the surface coating of the beads and appropriate blocking is important for assay performance. A major drawback of bead assays is that human serum components can directly bind to the beads and cause unspecific background. Serum pre-incubation with polyvinylalcohol, polyvinylpyrrolidone, and other proprietary reagents like Super ChemiBlock and Chemicon could reduce unspecific binding and resulted in increased S/N ratios [58]. Compared to planar microarrays, bead assays are not suited for the identification process of novel serum biomarkers due to the limited capture probe capacity. In a single microarray experiment up to 10,000 interactions can be simultaneously analyzed compared to bead assay with up to 500. However, bead assays are a versatile tool for routine application due to their easy automation and flexibility in experimental conditions. Novel serum biomarkers and predefined assay conditions can be easily implemented in a bead assay platform to determine the fine tuning of assay development.

The presented immunoassay platforms-microarrays and bead assays-enable a high throughput analysis with tiny amounts of patient sample. These endpoint measurements allow a yes/no answer in terms of analyte binding to the capture probe. However, the interplay between molecules *in vivo* is dynamic and does not follow a steady-state-equilibrium or adhere to a binding model. Therefore, a kinetic analysis that provides parameters like association and dissociation rates, as well as equilibrium constants, is sorely needed. These studies are of profound importance regarding the development of a diagnostic assay. Especially useful for optimizing the assay conditions, kinetic parameters are an indication helpful in defining the duration of incubation time and the washing stringency. Both parameters contribute to an improved assay performance while minimizing background noise, as well as non-specific binding to the target molecules. The kinetic measurement of antigen binding to antibody microspots on an array has been discussed by several reports. The conclusion of these reports is that mass transport dependency of the antibody-antigen microspot kinetic has been one of the main restrictions of array technology [31]. Although, the effects of microarray design parameters on microspot kinetics were theoretically and experimentally analyzed, the mass transport limitation still remains a serious problem. The optimization of parameters such as incubation vessel geometry, incubation time, stirring, capture probe density, spotting pattern, analyte concentration, *etc.* led to an improved sensitivity of kinetic microarrays. Nevertheless, mass transport limitations could not be entirely prevented to generate confidential kinetic parameters of the interaction process [59].

**Figure 4.**
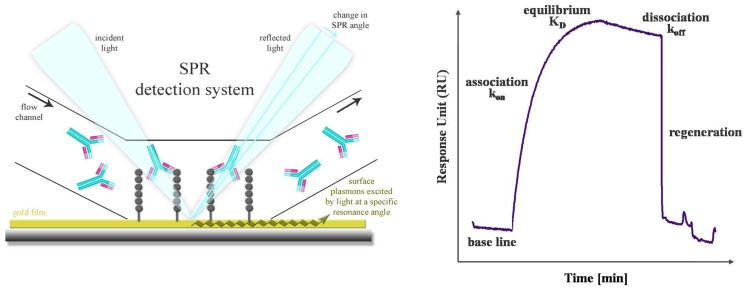
Detection system of SPR based Biacore Flexchip (**left**) and a sensorgram of a typical antibody-peptide interaction (**right**). The SPR effect and the expansion of plasmons along the sensor surface are caused by total reflection of light on the gold chip grating. Once the serum antibodies pass the surface and are bound to the immobilized probes the angle of reflection changes. These changes of SPR angle, that are proportional to mass absorption of the bound antibody, are visualized in real-time as Response Units (RU) in a sensorgram. The classical interaction process consists of baseline, association, equilibrium, dissociation, and regeneration.

SPR is a powerful technology to study label-free antibody-antigen interactions in real time. Particularly, high-content microarray-based biosensors are well suited for diagnostic screening of serum samples, epitope mapping and protein expression profiling [60]. The SPR phenomenon occurs when polarized light passes the interface of a high-refractive-index material (prism or grating) and a low-refractive-index medium (analyte). The light beam is completely reflected by a thin gold film. Another component, called the evanescence wave, passes into the gold film, where plasmons are excited. Thus, a resonant wave is transmitted and the intensity of the reflected light is decreased. Mass absorption by analyte binding to the capture probes on the gold surface leads to local refractive index changes. This causes a change in the SPR angle which is visualized as Response Units (RU). The progress of interaction is displayed in a sensorgram, a plot of response units against time (Figure 4). Conventional SPR systems have a few flow chambers, a maximum of four to six. As a result, only a limited number of analytes can be simultaneously measured. Serum screening is only possible if a small set of antigens is used. Nagel *et al.* restricted their SPR studies for serological detection of Lyme borrelioses to two widely used antigens. The whole proteins as well as two peptides, representing immunodominant domains, were used as capture probes [61]. But in general, different flow chambers have different antigens, and a direct comparison of kinetics and affinity ranking of more than four interactions is not practicable. De Boer *et al.* used a SPR platform that combines the microarray principle with SPR detection in one flow chamber. Thus, all SPR analyses were performed on a single array. The microarray contained 144 different glycans derived from the human parasite *Shistosoma mansoni* and was used for the simultaneous detection of glycan-specific serum antibodies [62]. Differences in the anti-glycan antibody repertoire from sera of *S. mansoni* infected patients, as well as uninfected controls were monitored. Moreover, differences between the antibody classes that responded to the infection were also revealed. A SPR biosensor was used to detect antibodies directly from human blood serum against the immunoreactive peptide epitope of EBV nuclear antigen. The results, yielded with this biosensor, were characterized in terms of reproducibility, detection limit and regeneration and compared with a conventionally used ELISA [63]. The reproducibility of the SPR sensor was obtained by using the standard deviation of the sensor response of different sensor chips and was determined in the range of 8 and 18% dependent on the serum antibody concentration. For the corresponding ELISA the inter-assay standard deviation was found to be between 5 and 20%. Thus, the reproducibility of the SPR sensor is comparable to that of an ELISA. The detection limit was estimated to be 0.1 ng/mL, which is lower by an order of magnitude than the detection limit of ELISA. The regeneration procedure was optimized and a minor loss of sensitivity was observed after 10 measurement cycles. This demonstrated the clinical relevance and the potential of the SPR sensor for usage in EBV diagnostics.

**Figure 5.**
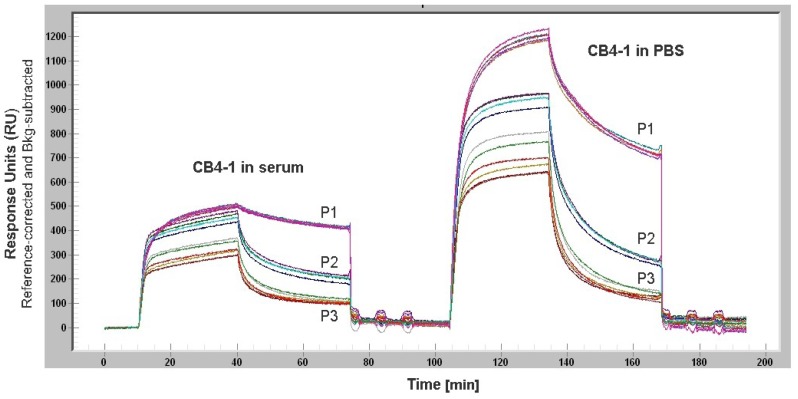
SPR study with Biacore Flexchip: Influence of complex media on binding behavior of CB4-1 antibody to its peptide epitopes. Biotinylated peptides (P1–P3), each in five replicates, were immobilized on a streptavidin coated gold chip. The binding of the monoclonal antibody CB4-1, either diluted in serum or PBS buffer, to the three different peptides is shown. Higher response units are achieved when CB4-1 is diluted in PBS buffer. Compared to that, the binding of CB4-1 diluted in serum is reduced by half. Based on the serum components the binding of CB4-1 to its peptides is affected and the signal of interest is masked by non-specific protein adsorption to the sensor surface.

Non-specific adsorption of proteins from complex media such as serum or cell lysate to the sensor surface causes a signal that masks the signal from the analyte of interest (Figure 5). This becomes detrimental for serum components like IgE or cytokines that are present in low concentrations. However, the study of Battaglia *et al.* demonstrated the detection of biologically relevant levels of the cytokine IL6 in cell culture media using a SPR sensor. For reducing the non-specific protein adsorption, the sensor surface was modified by a layer of NHS ester and 16-mercaptohexadecanoic [64]. Weinhart *et al.* suggested SAMs of linear polyglycerol derivates for gold surfaces. The study that focused on the protein adsorption to modified gold surfaces revealed that linear polyglycerol can be alternatively used for PEG as a protein resistant coating material [65].

Compared to microarrays and bead assays, label-free methods have the key advantage that additive detection antibodies or fluorescently labeled samples are not needed. Fluorescence detection requires the labeling of antibodies which is cost-intensive and may influence the bioactivity of the target molecules. The use of fluorescent dyes, either in patient samples or conjugated to detection antibodies, requires adapting the optimal S/N ratio to ensure a sensitive analysis with low background noise. Furthermore, an inefficient sample labeling interferes with the interaction of the target molecule and consequently leads to a loss of sensitivity. Another bottleneck is that the application of multiplex immunoassays assumes the use of high affinity antibodies, *i.e.*, approximately 10^11^–10^12^ M^−1^. All antibodies used in an assay should have closely comparable affinity constants to ensure the sensitivity of the test [23].

## 4. Conclusions

Nowadays, the awareness of the complexity of miscellaneous diseases is continuously increasing. Infection- or autoimmune diseases are influenced by various factors. Not an individual marker, but rather a panel of well-characterized marker molecules, defines the clinical picture, allowing a stratification of patients. When utilized for prognostics, the amount of molecules increases exponentially in order to study different disease states. Living up to the expectations of modern medicine makes the necessity of multi-parametric assays apparent. Multi-analyte immunoassays have the potential to be employed as the state-of-the-art technology for the in-depth serum biomarker discovery including validation studies and assay development. The performance strength of each immunoassay platform should be considered allowing their appropriate application in the process of biomarker discovery. Microarrays have the capacity for an unbiased and rapid serum screening to identify novel protein biomarkers from serum [66]. The development of a sensitive and specific microarray platform requires the choice of an appropriate surface chemistry, as well as the quantification of signal intensities, and the S/N ratio. To ensure the diagnostic significance of a protein biomarker a validation process is required. This profound validation process of the preselected biomarkers comprises an analysis concerning their specificity and sensitivity, and the definition of the assay conditions. Array-based SPR systems are exceedingly suited for this purpose since they provide kinetic data and allow a relatively high-throughput. The kinetics of the preselected interactions are analyzed by performing an affinity ranking and the molecules with the favored characteristics can be chosen for diagnostic assay. The validated molecules and the defined assay conditions are implemented in a laboratory friendly bead assay to reconsider the clinical utility. Biomarker candidates, that are inappropriate for clinical diagnostics can be excluded early on, before moving with that candidate in clinical trial.

## Figures and Tables

**Table 1.** Surface coatings for antigen microarrays. Adopted from [35].

**Surface coating**	**Immobilization mechanism**	**Chemical bounding**	**Advantages/Disadvatages**
Nitrocellulose	Hydrophobic adsorption	Non-covalent	No requirement for addit. coupling reagents nor antigen modification/weak binding, loss of activity, high S/N ratios
Poly-Lysine	Electrostatic Forces on charged surface	Non-covalent	
NHS ester	Lysine residues react with active ester to form amid bounds	Covalent	Stable interaction/unstable in aqueous solution, not orientated
Aldehyde	Primary amino-groups react with the aldehyde surface	Covalent	Stable interaction/not orientated
Epoxy	Nucleophilic residues (NH, SH) react with epoxy	Covalent	Stable to hydrolysis at neutral pH/not orientated
Streptavidin	Biotinylated residues bind to streptavidin	Strongest non-covalent bounding in nature 10^−14^ M	Site-specific immobilization, strong interaction/antigen modification
